# Common and Distinct Impacts of Autistic Traits and Alexithymia on Social Reward

**DOI:** 10.1371/journal.pone.0121018

**Published:** 2015-04-08

**Authors:** Lucy Foulkes, Geoffrey Bird, Elif Gökçen, Eamon McCrory, Essi Viding

**Affiliations:** 1 Department of Clinical, Educational and Health Psychology, University College London, London, United Kingdom; 2 MRC Social, Genetic and Developmental Psychiatry Centre, King's College London, London, United Kingdom; 3 Institute of Cognitive Neuroscience, University College London, London, United Kingdom; Harvard Medical School, UNITED STATES

## Abstract

According to the social motivation hypothesis of autism, individuals with high levels of autistic traits experience reduced levels of reward from social interactions. However, empirical evidence to date has been mixed, with some studies reporting lower levels of social reward in individuals with Autism Spectrum Disorder (ASD), and others finding no difference when compared to typically developing controls. Alexithymia, a subclinical condition associated with the reduced ability to identify and describe one’s own emotions, has been found to account for other affective difficulties observed inconsistently in individuals with ASD. The current study used a nonclinical sample (N = 472) to explore the associations between autistic traits and the value of six types of social reward, as measured by the Social Reward Questionnaire. In addition, we measured alexithymia to assess if this accounted for associations between autistic traits and social reward. There were three main findings. Firstly, higher levels of autistic traits were associated with significantly less enjoyment of admiration and sociability, and adding alexithymia to these models did not account for any additional variance. Secondly, both autistic traits and alexithymia were uniquely associated with reduced levels of enjoyment of prosocial interactions and sexual relationships. Thirdly, autistic traits were associated with higher levels of enjoyment of passivity and negative social potency, but these associations were no longer significant once alexithymia was taken into account, suggesting that co-occurring alexithymia accounted for these apparent associations. Overall, the current findings provide a novel and more nuanced picture of the relationship between autistic traits and social reward.

## Introduction

Autistic traits comprise communication and social interaction difficulties, as well as restrictive and repetitive behaviours or interests [1,2]. These traits are distributed continuously in the normal population, and individuals with a diagnosis of Autism Spectrum Disorder (ASD) have high levels of impairment in all areas of difficulty [3].

The social motivation hypothesis of autism posits that individuals with ASD experience less social reward than their typically developing peers (e.g. [4–5]). Social reward can be defined as the motivational (‘wanting’) and pleasurable (‘liking’) aspects of interacting with others [6], and there is evidence that these processes are impaired in individuals with ASD. For example, children with ASD score significantly lower than their peers on the Wish for Social Interaction Scale, which asks respondents how much they would like to chat with or play with people shown in a series of photographs [7]. Similarly, individuals with ASD reported less pleasure from social activities when compared to controls [8–9]. There is also support for the social motivation hypothesis from neuroimaging studies: individuals with ASD have shown reduced neural activation in frontostriatal regions implicated in reward processing during social reward tasks relative to typically developing controls (reward anticipation: [10]; receipt: [11]; learning: [12]). An EEG study found that children with ASD showed reduced event-related potentials while anticipating and receiving social rewards compared with typically developing controls ([13]).

However, the theory that individuals with ASD experience less social reward is not wholly supported by the empirical evidence. For example, compared to typically developing controls, children with ASD are equally motivated to use an effortful keypress sequence or an implicit joystick-based paradigm to view faces, a common social reward (keypress sequence: [14]; joystick paradigm: [7]). In a Go/No-Go task, children with ASD responded with *faster* reaction times to social reward than typically developing controls [15]. In addition, endogenous opioids play a key role in the hedonic value of social (and other) experiences, but there is little support that levels are decreased in individual with ASD [16–17].

A possible explanation for the mixed findings thus far is that ASD is a heterogeneous disorder, with individuals presenting with different types and severities of difficulties under the same umbrella diagnosis [2,18]. It has also long been recognised that individuals with ASD can vary considerably in terms of their motivation to interact with others [19]. It is therefore possible that across individuals with high levels of autistic traits, there are considerable individual differences in the experience of social motivation and reward. As such, it is critical to consider additional constructs that can co-occur with autistic traits, which may provide explanatory power for the mixed results of social reward in ASD found to date.

A construct that has received substantial attention in recent years is alexithymia, a subclinical condition associated with difficulties in identifying and describing one’s own emotions [20]. Alexithymia is reported in approximately 10% of the general population [21], but occurs at significantly elevated levels in many psychiatric conditions, including anorexia nervosa [22], depression [23] and schizophrenia [24]. Of particular relevance, elevated levels of alexithymia occur in approximately 50% of individuals with ASD [25]. Recent research has demonstrated that alongside deficits in processing their own emotions, individuals with alexithymia also have difficulties with processing *others’* emotions, such that these individuals have difficulty recognising others’ facial expressions [26–29] and have reduced levels of empathy [30–31]. Such emotion processing difficulties have been found inconsistently in individuals with ASD, and recent research indicates that once co-occuring alexithymia is taken into account, a number of apparent associations between autistic traits and affective processing deficits disappear [25,28,30]. This suggests that deficits in affective processing previously attributed to autism may in fact be explained by the co-occurrence of elevated levels of alexithymia.

Reward processing is, in part, a form of affective processing [32]. In particular, social reward processing implicates the generation and representation of emotional states in relation to others (e.g. [33]). Since alexithymia appears to play a role in other affective processing deficits associated with autistic traits [25], it may be important to consider alexithymia when assessing autistic traits and social reward processing. Alexithymia may explain, at least in part, why the negative association between autistic traits and social reward is sometimes observed (e.g. [4–5]). Indeed, studies that have focused on alexithymia (but not their co-occurrence with ASD) have found that alexithymia is significantly associated with social anhedonia [34] and with lower levels of satisfaction in intimate relationships [35], suggesting that alexithymia does negatively impact the experience of social reward.

The current study seeks to understand two main questions. First, are elevated levels of autistic traits associated with reduced levels of social reward? Second, does alexithymia account for any observed associations between autistic traits and social reward? To measure social reward, we used the Social Reward Questionnaire [36], which indexes the level of six subtypes of social reward: admiration; negative social potency; passivity; prosocial interactions; sexual relationships; and sociability. This allowed us to explore in more detail the specific *type* of social reward value that may be associated with autistic traits, a question that to our knowledge has not yet been addressed. Autistic traits were measured with the Autism Spectrum Quotient [3], and alexithymic traits with the Toronto Alexithymia Scale [37].

We made four specific hypotheses. Firstly, we hypothesised that the association between autistic traits and the enjoyment of admiration would be negative, due to the lower levels of impression management seen in this population [38–39]. Secondly, we hypothesised that autistic traits would be negatively associated with enjoyment of prosocial interactions, as a recent study found that individuals with high levels of autistic traits reported less personal satisfaction when required to choose a prosocial course of action [40]. Thirdly, we hypothesised that there would be a negative association between autistic traits and enjoyment of sociability. This subscale measures the enjoyment of socialising, such as spending time with others and going to parties, and engagement in this type of activity is rare in individuals with ASD [41]. Finally, we hypothesised that the association between autistic traits and alexithymia would be positive, in line with existing research [25, 42].

The rest of our analyses were exploratory. We made no specific hypothesis regarding the association between autistic traits and enjoyment of sexual relationships because the existing research on this subject has been mixed. For example, two reviews concluded that individuals with autism *are* interested in sexual relationships with others, but that they tend to have less actual sexual experiences and more problematic sexual behaviours [43–44], leaving it unclear how autistic traits might be associated with sexual reward. Our analysis of the passivity and negative social potency subscales of the SRQ was also exploratory as there has been limited previous research on this topic, although one study found that adolescents with ASD did not differ from controls with regard to frequency or duration of submissive/passive behaviours towards peers [45], suggesting there may be no association between autistic traits and passivity. Our final exploratory analyses relates to the role of alexithymia. We wanted to investigate whether any associations between autistic traits and social reward are accounted for by co-occurring alexithymic traits, as appears to be the case for the relationship between autistic traits and other kinds of affective processing [25], but make no specific hypotheses regarding which type of social reward.

## Method

### Ethics Statement

All participants provided online informed consent and the study was approved by the University College London Clinical, Educational and Health Psychology Research Ethics committee.

### Participants

Participants (N = 472; 290 female, 182 male) were recruited via Amazon’s online crowd-sourcing website Mechanical Turk (MTurk; www.mturk.com). MTurk is an international online platform that allows researchers to post tasks or questionnaires that participants complete in return for payment, and is a cost-effective source of valid and reliable questionnaire data [46]. In the current study, participants signed up via MTurk and then completed the questionnaires using the online survey software LimeSurvey (www.limesurvey.org).

Participants were aged 18 to 65 years old (mean 35.4, SD 12.1) and all lived in the United States. The ethnicity of the sample was as follows: 76.42% White, 10.0% Black, 5.5% Asian, 3.6% Hispanic, and 4.4% mixed or other ethnicities. The highest completed education level was as follows: 36.2% Bachelors degree, 25.6% College, 24.8% High school, 13.3% Postgraduate degree. A total of 480 participants completed the questionnaires, but five submissions were excluded because the participant had taken part twice (second submission excluded) and three submissions were excluded on the grounds of poor data quality (i.e. the participant answered with the highest response option for all items in all three questionnaires). This left a final sample of N = 472.

### Questionnaires

#### Autistic traits

Autistic traits were measured using the Autism Spectrum Quotient (AQ; [3]), a 50-item questionnaire measuring autistic traits in adults of normal intellectual ability. Participants indicate how much they agree with each item on a scale of 1 to 4 (1 = Strongly disagree, 4 = Strongly agree), with 26 of the items reverse-coded. The current paper uses the original dichotomous coding scheme, in which ‘Strongly agree’ or ‘Agree’ responses are awarded one point, which are then summed to form a total score (‘Strongly disagree’ or ‘Disagree’ responses are awarded no points; [3]). Respondents can therefore score in the range of 0 to 50, with higher scores reflecting more severe ASD symptomatology. The AQ has good psychometric properties [3] and in the current sample Cronbach’s Alpha scores also indicated good reliability (Cronbach’s Alpha. 78).

#### Social reward

The Social Reward Questionnaire (SRQ; [36]) is a 23-item scale used to measure individual differences in the value of social rewards. Each item begins “I enjoy” and then describes a different type of social interaction. Participants are asked to consider the item in relation to all their social interactions, e.g. friends, partners, family, colleagues or people they have just met. Responses are given on a 1 to 7 scale (1 = Disagree strongly, 7 = Agree strongly).

The SRQ consists of six subscales, each representing a domain of social reward. *Admiration* measures the enjoyment of being flattered, liked and gaining positive attention; *Negative Social Potency* measures the enjoyment of being cruel, callous and using others for personal gains; *Passivity* measures the enjoyment of giving others control and allowing them to make decisions; *Prosocial Interactions* measures the enjoyment of having kind, reciprocal relationships; *Sexual Relationships* measures the enjoyment of having frequent sexual experiences; and *Sociability* measures the enjoyment of engaging in group interactions.

The SRQ has good psychometric properties [36] and in the current sample Cronbach’s Alpha scores indicated good reliability (Admiration. 88, Negative Social Potency. 86, Passivity. 82, Prosocial Interactions. 84, Sexual Relationships. 84, Sociability. 78).

#### Alexithymia

Alexithymia was measured with the 20-item Toronto Alexithymia Scale (TAS-20; [37]). Participants rate items on a scale of 1–5 (1 = I strongly disagree, 5 = I strongly agree). The TAS-20 has good psychometric properties [37] and a Cronbach’s Alpha of. 88 in the present study also indicated good reliability.

## Results

### Descriptives and correlations

Descriptives for all three questionnaires measures (AQ, TAS-20 and SRQ) are shown in Table 1 (see also S1 Table for descriptives for males and females separately, and S2 Table for numbers of participants meeting cut-off scores for the AQ [63] and TAS-20). Pearson and Spearman correlational analyses (as appropriate depending on the normality of the bivariate residuals) were conducted using IBM SPSS Statistics 20.0 for Windows. Correlations between autistic traits (as measured by total AQ score), alexithymia (as measured by total TAS-20 score) and social reward subscales (as measured by SRQ) are shown in Table 2. Partial correlations are presented, with age and gender controlled for in all correlations. Benjamini and Hochberg False Discovery Rate [47] was used to control for the probability of making a Type I error on multiple comparisons, and only corrected p-values are presented.

**Table 1:** Descriptives for all questionnaire measures.

	Minimum	Maximum	Mean (SD)
Autistic traits	5.00	42.00	19.61 (6.64)
Alexithymia	20.00	83.00	45.99 (13.02)
Social reward			
*Admiration*	1.00	7.00	5.10 (1.32)
*Negative Social Potency*	1.00	6.20	2.05 (1.12)
*Passivity*	1.00	6.67	3.03 (1.37)
*Prosocial Interactions*	2.20	7.00	5.97 (0.92)
*Sexual Relationships*	1.00	7.00	4.66 (1.71)
*Sociability*	1.00	7.00	4.48 (1.46)

N = 472

N.B. Autistic traits measured by total AQ score; alexithymia measured by total TAS-20 score; social reward measured by total SRQ subscale scores

**Table 2:** Correlations between autistic traits, alexithymia and social reward.

	Autistic traits	Alexithymia
Social reward		
*Admiration*	-.25**	-.18**
*Negative Social Potency*	.17** ^a^	.41**
*Passivity*	.11*	.24**
*Prosocial Interactions*	-.33** ^a^	-.42**
*Sexual Relationships*	-.20**	-.18**
*Sociability*	-.58**	-.28**
Alexithymia	.51**	

N = 472.

*p<.05

**p<.01

Age and gender partialled out. Only corrected p values are shown

^a^Partial Spearman correlations are reported due to nonnormality of data. All others reported are partial Pearson correlations

N.B. Autistic traits measured by total AQ score; alexithymia measured by total TAS-20 score; social reward measured by total SRQ subscale scores

### Regressions

A series of regressions were run, modelling the variance in six social reward subtypes: admiration, negative social potency, passivity, prosocial interactions, sexual relationships and sociability (see Table 3). Age and gender were entered in Step 1 of each model, autistic traits were entered in Step 2, and alexithymia was entered in Step 3 using the enter method in SPSS.

**Table 3:** Results from hierarchical regression models predicting subtypes of social reward.

	Admiration	Negative Social Potency	Passivity	Prosocial Interactions	Sexual Relationships	Sociability
*Step 1*						
Age	-.18**	-.31**	-.30**	.12**	-.12*	-.19**
Gender	.02	-.27**	.06	.23**	-.25**	.04
*Step 2*						
Age	-.21**	-.29**	-.28**	.08	-.14**	-.26**
Gender	.00	-.26**	.07	.21**	-.26**	.00
AQ	-.25**	.15**	.11*	-.31**	-.20**	-.58**
*Step 3*						
Age	-.22**	-.25**	-.26**	.04	-.15**	-.26**
Gender	-.01	-.22**	.09*	.18**	-.27**	.01
AQ	-.21**	-.05	-.02	-.13**	-.15**	-.59**
TAS-20	-.07	.40**	.24**	-.35**	-.10^	.03

N.B. Standardized coefficients are shown.

^p<.1

*p<.05.

**p<.01.

**Admiration:** Step 1 *R*2 = 3.2%, Step 2 *R*2 = 9.1% (Δ*R*2 = 6.0% *p*<.01), Step 3 *R*2 = 9.5% (Δ*R*2 = 0.3%, p = .20).

**Negative Social Potency:** Step 1 *R*2 = 19.4%, Step 2 *R*2 = 21.6% (Δ*R*2 = 2.3% *p*<.01), Step 3 *R*2 = 32.8% (Δ*R*2 = 11.2%, p<.01).

**Passivity:** Step 1 *R*2 = 8.5%, Step 2 *R*2 = 9.6% (Δ*R*2 = 1.0%, *p*<.05), Step 3 *R*2 = 13.7% (Δ*R*2 = 4.1%, p<.01).

**Prosocial Interactions:** Step 1 *R*2 = 7.6%, Step 2 *R*2 = 16.9% (Δ*R*2 = 9.3%, *p*<.01), Step 3 *R*2 = 25.5% (Δ*R*2 = 8.6%, p<.01).

**Sexual Relationships:** Step 1 *R*2 = 8.3%, Step 2 *R*2 = 12.0% (Δ*R*2 = 3.8%, *p*<.01), Step 3 *R*2 = 12.7% (Δ*R*2 = 0.6%, p = .06).

**Sociability:** Step 1 *R*2 = 3.5%, Step 2 *R*2 = 36.1% (Δ*R*2 = 32.6%, *p*<.01), Step 3 *R*2 = 36.2% (Δ*R*2 = 0.1%, p = .47).

### Autistic traits

When added to the demographic variables (Step 2), autistic traits were a significant predictor of all six social reward subtypes (admiration: β = -.25, t(471) = -5.55, p<.01; negative social potency: β = .15, t(471) = 3.68, p<.01; passivity: β = .10, t(471) = 2.33, p<.05; prosocial interactions: β = -.31, t(471) = -7.24, p<.01; sexual relationships: β = -.20, t(471) = -4.47, p<.01; sociability: β = -.58, t(471) = -15.46, p<.01). This step significantly improved the fit of all six models (admiration: variance accounted for increased by 6.0%, F(1,468) = 30.79, p<.01; negative social potency: variance accounted for increased by 2.3%, F(1,468) = 13.54, p<.01; passivity: variance accounted for increased by 1.0%, F(1,468) = 5.41, p<.05; prosocial interactions: variance accounted for increased by 9.3%, F(1,468) = 52.34, p<.01; sexual reward: variance accounted for increased by 3.8%, F(1,468) = 19.96, p<.01; sociability: variance accounted for increased by 32.6%, F(1,468) = 238.97, p<.01).

### Alexithymia

When alexithymia was added to each model (Step 3), this variable was a significant predictor of four social reward subtypes: negative social potency, passivity, prosocial interactions and, at trend level, sexual relationships (negative social potency: β = .40, t(471) = 8.81, p<.01; passivity: β = .24, t(471) = 4.73, p<.01; prosocial interactions: β = -.35, t(471) = -7.34, p<.01; sexual reward: β = -.10, t(471) = -1.86, p = .06). Adding alexithymia significantly improved the fit of these four models, although only at trend level for sexual relationships (negative social potency: variance accounted for increased by 11.2%, F(1,467) = 77.64, p<.01; passivity: variance accounted for increased by 4.1%, F(1,467) = 22.40, p<.01; prosocial interactions: variance accounted for increased by 8.6%, F(1,467) = 53.87, p<.01; sexual reward: variance accounted for increased by 0.6%, F(1,467) = 3.47, p = .06). Adding alexithymia at this step caused the autistic traits predictor to lose significance in the negative social potency and passivity models. This suggests that autistic traits account for very little variance in reward from negative social potency or passivity once alexithymia has been accounted for. Alexithymia and autistic traits both uniquely accounted for variance in models predicting reward from prosocial interactions and sexual relationships (autistic traits were associated at trend level for sexual relationships).

When added at Step 3, alexithymia was not a significant predictor of admiration or sociability (admiration: β = -.07, t(471) = -1.29, p = .20; sociability: β = .03, t(471) = .73, p = .47). This step did not improve the fit of these two models (admiration: variance accounted for increased by 0.3%, F(1,467) = 1.66, p = .20; sociability: variance accounted for increased by 0.1%, F(1,467) = .53, p = .47).

## Discussion

The current study explored associations between autistic traits and different types of social reward, and assessed whether co-occurring alexithymia might explain some of these associations. There were three main findings. Firstly, autistic traits were associated with lower levels of enjoyment of admiration and sociability, and adding alexithymia to these models did not account for any additional variance in these types of social reward. Secondly, both autistic traits *and* alexithymia were uniquely negatively associated with the enjoyment of prosocial interactions and sexual relationships. Finally, autistic traits were associated with higher levels of enjoyment from passivity and negative social potency, but these associations were no longer significant once alexithymia was entered into the models, suggesting that it is alexithymia and not autistic traits that is associated with elevated levels of these rewards. Together, these findings give a novel and nuanced picture of the relationship between autistic traits and social reward.

The negative associations found here are in line with existing evidence of atypical social functioning in autism and alexithymia. For example, the negative association between autistic traits and enjoyment of admiration is in keeping with evidence that individuals with ASD engage in less impression management than controls (that is, they are less likely to attempt to influence others’ opinion of themselves) [38–39]. There is some evidence that individuals with ASD do engage in impression management when they are sufficiently motivated to do so [48]. The current findings complement this by suggesting that perhaps external motivation is necessary to engage in impression management because individuals with ASD experience attenuated intrinsic social reward from this behaviour. The negative association between autistic traits and enjoyment of sociability fits with evidence that individuals with ASD are less likely to engage in casual socialising compared to their peers [41]. The negative associations observed between both autistic traits and alexithymia and enjoyment of prosocial interactions support previous evidence that both constructs are associated with less prosocial behaviour (autistic traits: [40]; alexithymia: [49]). Finally, the negative associations between both autistic traits and alexithymia and enjoyment of sexual relationships are in line with findings that individuals with ASD have fewer sexual experiences than their peers [44] and experience higher levels of physical anhedonia [9], and that alexithymia is associated with lower levels of sexual satisfaction [35,50]. In all, the negative associations in the current study are in keeping with extant data suggesting that both autistic traits and alexithymia are associated with a reduction in socially-oriented behaviour.

The apparent positive associations between autistic traits and passivity/negative social potency were driven by co-occurring alexithymia. We contend that the positive association between alexithymia and enjoyment of passivity makes intuitive sense. The passivity subscale assesses the enjoyment of social interactions in which someone else takes the lead, with items such as ‘I enjoy someone else making decisions for me’ and ‘I enjoy following someone else’s rules’ [36]. Since alexithymia has been associated with difficulty in interpreting social signals (e.g. [26–27, 28]), it seems reasonable to propose that individuals with high levels of alexithymia may enjoy interactions in which their partner takes a facilitative, leading role.

The positive association between alexithymia and enjoyment of negative social potency is more difficult to interpret. This subscale contains items that measure enjoyment of cruel, vindictive behaviour towards others, such as ‘I enjoy making someone angry’ and ‘I enjoy tricking someone out of something’ [36]. One possible explanation for the positive association between alexithymia and this social reward subscale is that behaving cruelly towards others produces a very predictable and explicit emotional response in others. As social cues can be difficult to interpret for individuals with high levels of alexithymia [26–28], it may be that the predictability of response that cruel behaviour elicits is rewarding for alexithymic individuals. An alternative explanation could be that the reduced levels of empathy in alexithymia [30–31,51] facilitate the enjoyment of negative social potency. Specifically, it may be that for most individuals, typical levels of empathy ensure that behaving cruelly towards others is aversive. However, if empathy levels are reduced, as they are in alexithymia, it may be that such interactions are not only not aversive but actually enjoyable. Psychopathic traits may also be relevant, as these have been associated with both alexithymia [21] and the enjoyment of cruelty [6]. These interpretations are speculative and further research is necessary to better understand the relationship between alexithymia and enjoyment of negative social potency. For example, paradigms that manipulate the predictability and valence of emotional response in a social interaction partner, and measure levels of empathy and psychopathic traits in the instigator, could help to identify why negative social interactions might be reported as enjoyable for individuals with alexithymia.

Overall, the current data allow interesting speculation with regard to the processes that might be involved in typical social reward processing. For example, autistic traits but not alexithymia were associated with reduced enjoyment of admiration and sociability. We speculate that, for these types of social reward, processes that are specifically associated with autism may be important. For example, theory of mind, the ability to represent another person’s mental states, is impaired in autism [52]. Based on the current findings, we speculate that an intact theory of mind may be important for enjoying the admiration of others and socialising with others. This makes intuitive sense with admiration, in which the capacity to represent what others think (of oneself) seems particularly relevant. In contrast, the current data suggest that for the enjoyment of prosocial interactions and sexual relationships, processes that are impaired in both autism *and* alexithymia may be important. Specifically, the impaired theory of mind seen in autism and the deficits in describing one’s own and others’ emotions seen in alexithymia may both contribute to a reduced ability to experience reward from prosocial interactions or sexual relationships. Finally, for the enjoyment of passivity and negative social potency, we speculate that processes impaired in alexithymia, rather than autism, may facilitate or enable enjoyment from these types of social interactions. We fully acknowledge the speculative nature of these interpretations, and recognise the need for further research to identify the processes that enhance or impair enjoyment of different social rewards. For example, tasks that specifically measure different affective and cognitive processes that are impaired in autism and alexithymia could be administered alongside the Social Reward Questionnaire to see how these processes are associated with the enjoyment of different types of social reward.

There are also additional constructs not measured in the current study that may be important in fully understanding the relationship between autistic traits, alexithymia and social reward. For example, there are elevated levels of anxiety and depression in both autistic (anxiety: [53]; depression: [54]) and alexithymic (anxiety: [55]), depression: [23]) populations, and anxiety and depression are both associated with atypical social reward processing (anxiety: [56]; depression: [57]). Therefore, it may be that some of the associations reported here between autistic traits and/or alexithymia and subtypes of social reward are mediated or moderated by levels of anxiety and depression. This is an important avenue of future research to explore in order to fully understand the pattern of associations reported here between autistic traits, alexithymia and social reward.

It is important to emphasise that participants in this study were from a community, not clinical, population. This limits the generalizability of the current findings to those with a clinical diagnosis of ASD. At sufficiently high levels (i.e. those that warrant a diagnosis), autistic traits may be qualitatively different to those at lower levels, or the relationship between autistic traits, social reward and alexithymia may be different. However, it should be noted that in the current sample, 18 participants scored 32 or higher on the AQ—an approximate threshold proposed by the original authors of the AQ for discriminating individuals with and without a clinical diagnosis of ASD ([3]; see also S2 Table). This suggests that the current findings would hold within a clinical sample, although further research is required. Specifically, research that asks similar questions to that of the current study, but in clinical groups, is critical to fully delineate the relationship between these constructs.

In general, the presented evidence that individuals with high levels of autistic traits enjoy social interactions less should be interpreted with caution. Autistic traits have also been associated with elevated levels of loneliness [58–59], and loneliness implies a desire for interactions or relationships that goes unfulfilled. This suggests that perhaps individuals with ASD do want social relationships; they just often do not have the skills to succeed at them. It may be that their lifelong difficulty interpreting social signals has made social exchanges fraught with confusion and complexity, and it is this which leads them to report less enjoyment of social interactions [58–61]. Similarly, the negative association between autistic traits and enjoyment of sexual relationships reported here could be interpreted as a dislike of the complex rules of dating and romantic relationships associated with sex, as opposed to a lack of reward value of sex itself [62]. It is a key goal of future research to identify in more detail why individuals with high levels of autistic traits report reduced enjoyment of social interactions, and what might facilitate positive relationships in this population.

## Conclusion

In conclusion, the current study presents novel evidence exploring the relationship between autistic traits, alexithymia and six types of social reward. There were three main findings: first, autistic traits (but not alexithymia) were associated with reduced levels of enjoyment of admiration and sociability; second, autistic traits and alexithymia were both associated with reduced levels of enjoyment of prosocial interactions and sexual relationships; and third, alexithymia (but not autistic traits) was associated with increased levels of enjoyment of passivity and negative social potency. These results provide a detailed picture of reduced social reward in individuals with high levels of autistic traits, which are worthy of further exploration in clinical samples.

## Supporting Information

S1 Dataset File containing all data underlying findings described in manuscript. (XLS)

S1 Table Descriptives for males and females for all questionnaire measures. (DOCX)

S2 Table Numbers of participants in cut-off categories for the AQ and TAS-20. (DOCX)
