# Supplementary material for: Common and Distinct Impacts of Autistic Traits and Alexithymia on Social Reward
Source: PLoS One. 2015 Apr 8;10(4):e0121018. doi: 10.1371/journal.pone.0121018 (PMC4390314; doi:10.1371/journal.pone.0121018)
Supplement: S1 Table — (DOCX) [file pone.0121018.s002.docx]

**S1 Table.** Descriptives for males and females for all questionnaire measures

|  | **Males (N=182)** | | | **Females (N=290)** | | |
| --- | --- | --- | --- | --- | --- | --- |
|  | Minimum | Maximum | Mean (SD) | Minimum | Maximum | Mean (SD) |
| Autistic traits | 5.00 | 36.00 | 20.35 (6.78) | 5.00 | 42.00 | 19.15 (6.53) |
| Alexithymia | 20.00 | 80.00 | 48.53 (13.48) | 20.00 | 83.00 | 44.39 (12.48) |
| Social reward |  |  |  |  |  |  |
| *Admiration* | 1.00 | 7.00 | 5.12 (1.27) | 1.00 | 7.00 | 5.09 (1.35) |
| *Negative Social Potency* | 1.00 | 6.20 | 2.50 (1.21) | 1.00 | 5.60 | 1.77 (0.96) |
| *Passivity* | 1.00 | 6.67 | 3.01 (1.36) | 1.00 | 6.67 | 3.05 (1.37) |
| *Prosocial Interactions* | 2.20 | 7.00 | 5.68 (1.04) | 2.80 | 7.00 | 6.15 (0.80) |
| *Sexual Relationships* | 1.00 | 7.00 | 5.23 (1.59) | 1.00 | 7.00 | 4.30 (1.69) |
| *Sociability* | 1.00 | 7.00 | 4.46 (1.49) | 1.00 | 7.00 | 4.49 (1.46) |

Total N=472. N.B. Autistic traits measured by total AQ score; alexithymia measured by total TAS-20 score; social reward measured by total SRQ subscale scores
