# Supplementary material for: Common and Distinct Impacts of Autistic Traits and Alexithymia on Social Reward
Source: PLoS One. 2015 Apr 8;10(4):e0121018. doi: 10.1371/journal.pone.0121018 (PMC4390314; doi:10.1371/journal.pone.0121018)
Supplement: S2 Table — (DOCX) [file pone.0121018.s003.docx]

**S2 Table.** Numbers of participants in cut-off categories for the AQ and TAS-20

|  | **Males (N=182)** | **Females (N=290)** | **Total (N=472)** |
| --- | --- | --- | --- |
| **AQ** |  |  |  |
| *Autism phenotypes^1^* |  |  |  |
| Typical (AQ total = 0-22) | 109 | 208 | 317 |
| Broad (23-28) | 54 | 58 | 112 |
| Medium (29-34) | 17 | 19 | 36 |
| Narrow (35-50) | 2 | 5 | 7 |
| *AQ cut-off scoring^2^* |  |  |  |
| AQ total = 32-50 | 10 | 8 | 18 |
|  |  |  |  |
| **TAS-20** |  |  |  |
| *TAS-20 cut-off scoring^3^* |  |  |  |
| Non-alexithymia (TAS-20 total = 20-51) | 101 | 210 | 311 |
| Possible alexithymia (52-60) | 41 | 46 | 87 |
| Alexithymia (61-100) | 40 | 34 | 74 |

^1^Cut-off criteria taken from reference [63] in manuscript

^2^Cut-off criteria taken from reference [3] in manuscript

^3^Cut-off criteria taken from reference [37] in manuscript

NB. No cut-off scoring system exists for the Social Reward Questionnaire.
